# The Use of Empagliflozin Post Myocardial Infarction

**DOI:** 10.7759/cureus.40602

**Published:** 2023-06-18

**Authors:** Kapilraj Ravendran, Nikolaos Madouros, Edzhem Yoztyurk, Aishwarya Wilson, Maria J Jeejo, Monica E Camelio, Akatya Sinha, Ananya George, Mriganka Rai, Hussain K Malik

**Affiliations:** 1 General Surgery, East Sussex Healthcare NHS Trust, Brighton and Hove, GBR; 2 Medicine, Gradscape, London, GBR; 3 Surgery Working Group, Society of Junior Doctors, Athens, GRC; 4 Internal Medicine, Medical University of Sofia, Sofia, BGR; 5 Vascular Surgery, St George's University Hospital, London, GBR; 6 Medicine, MGM (Mahatma Gandhi Mission) Medical College, Mumbai, IND; 7 Gastroenterology, Norfolk and Norwich University Hospital, Norwich, GBR

**Keywords:** sodium-glucose cotransporter-2, left ventricular ejection fraction, diabetes, lvef (left ventricular ejection fraction), myocardial infarction, sodium-glucose cotransporter-2 (sglt-2) inhibitors, empagliflozin

## Abstract

Empagliflozin is a sodium-glucose cotransporter 2 (SGLT2) inhibitor that is mainly used for the treatment of type 2 diabetes mellitus. The study's objective was to assess empagliflozin's effects and impacts on post-myocardial patients to highlight its worth in comparison to alternative therapies. Only studies evaluating the effects of empagliflozin on individuals who have undergone a myocardial infarction (MI) are included in this review of the literature, which employed PubMed, Google Scholar, and Embase. To compare the advantages of empagliflozin for individuals who have recently experienced a myocardial infarction, abstracts from pertinent articles were retrieved, and complete publications were reviewed. A total of four articles were reviewed, which showed that in patients who suffered from a recent MI, empagliflozin caused a significant decrease in N-terminal pro-brain natriuretic peptide (NT-proBNP). Additionally, it was shown that these individuals had better echocardiographic results for both structural and functional metrics. With studies showing a significantly larger median NT-proBNP decrease with empagliflozin compared to placebo among patients hospitalised with an acute big MI when empagliflozin was started early and administered in addition to the post-MI care suggested by guidelines, it is safe to say that the benefits outweigh the risks. There are currently larger double-blind trials in progress to prove the hypothesis of the benefits of empagliflozin for post-MI patients.

## Introduction and background

Sodium-glucose cotransporter 2 (SGLT2) inhibitors prevent heart failure hospitalisation in high-risk diabetics who typically do not have heart failure [1]. It has been shown that taking dapagliflozin or empagliflozin reduces the risk of cardiovascular death or heart failure hospitalisations in patients with heart failure who have an ejection fraction of 40% or less [1,2]. Sotagliflozin was found to reduce the incidence of heart failure hospitalisation in small subgroups of diabetic patients with heart failure who had an ejection fraction of 50% or above [1,2]. Empagliflozin was discovered to reduce the incidence of cardiovascular death or heart failure hospitalisations in individuals with heart failure who had maintained ejection fraction in a recent large definitively powered trial [1].

SGLT2 inhibitors primarily affect kidney SGLT2, which eliminates extra glucose [2]. Yet, by an unidentified mechanism unrelated to glucose, they also considerably lower cardiovascular mortality and the hospitalisation rate for heart failure [2]. Given that cardiomyocytes do not express SGLT2, it is necessary to determine if the medication directly affects the heart to induce cardioprotection and to understand the associated direct protective molecular pathways [2]. Current theories on how SGLT2 inhibitors protect the heart imply that autophagy activation may be the mechanism [3].

SGLT2 inhibition has cardioprotective effects such as diuresis and natriuresis, blood pressure reduction, erythropoiesis, improved cardiac energy metabolism, reduced inflammation, suppression of the sympathetic nervous system, prevention of unwanted cardiac remodelling, and prevention of ischemia and reperfusion [4,5].

Recent clinical studies showed that SGLT2 inhibitor class of anti-diabetic medications had positive cardiovascular effects, including a decrease in cardiovascular death, non-fatal myocardial infarction (MI), heart failure, and non-fatal stroke, as well as all-cause mortality [6].

The study's objective was to assess empagliflozin's effects and impacts on post-myocardial patients to highlight its advantages. Empagliflozin received FDA clearance for an indication that includes lowering the risk of cardiovascular mortality in individuals with type 2 diabetes and existing cardiovascular disease. The study is a review of published cases of empagliflozin treatment in post-myocardial patients. We are aware of very little research on humans, with two large blind studies in progress.

## Review

Materials and methods

**Figure 1:**
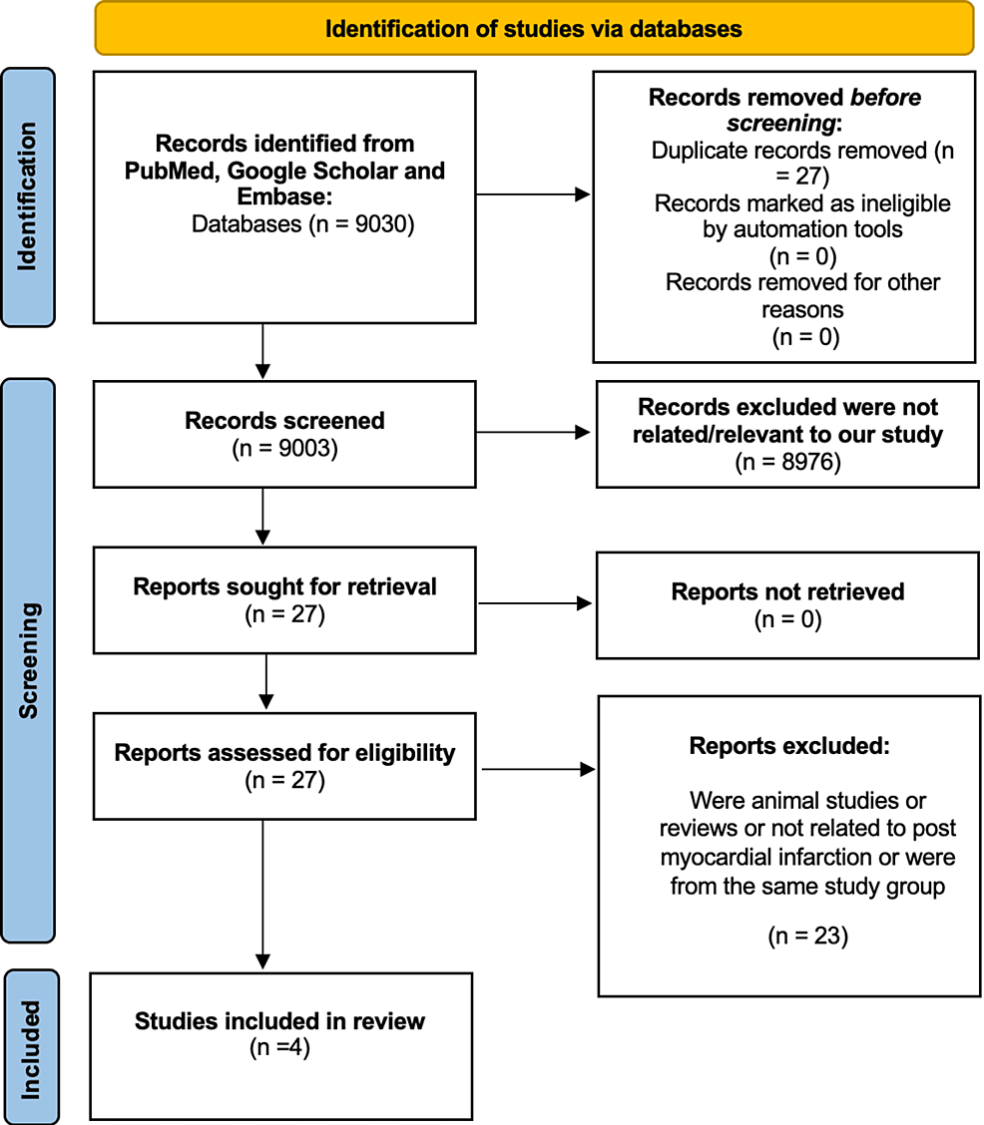
PRISMA flow diagram demonstrating the literature selection strategy PRISMA: Preferred Reporting Items for Systematic Reviews and Meta-Analyses.

This is a literature review where articles were searched and reviewed using PubMed, Google Scholar, and Embase. The keywords "empagliflozin" or "SGLT2 inhibitors" were used along with the Medical Subject Headings (MeSH) "post myocardial infarction". To compare the advantages of empagliflozin in post-MI patients, abstracts from every paper were retrieved, and the entire texts of each were read and taken into consideration. Six authors searched English titles and abstracts for pertinent studies. Our review included data that were abstracted from each study and linked to the use of empagliflozin after MI. Our article does not include any animal experiments. A total of 9.030 items in all were found. Between the various databases, 27 duplicate articles were eliminated. Only 27 papers remained after the screening, which involved looking through the titles, abstracts, and entire articles. A total of 23 more papers were disregarded because they either originated from the same research or concentrated mostly on other illnesses, including diabetes and the heart. Only case reports and case reviews were used in our study. We eliminated all literature reviews from the analysis. Following an extensive examination, four pertinent studies were included. The Preferred Reporting Items for Systematic Reviews and Meta-Analyses (PRISMA) flowchart supplied shows that a literature search was done and the results are presented in Figure 1 [7].

Results

After excluding secondary sources from the database search and eliminating duplicates, the initial search turned up 27 pertinent articles. After removing 23 records due to their lack of relevance to the issue after screening titles and abstracts (n = 27), the complete texts of four publications were evaluated. The studies in total had 669 patients with post-myocardial, of which 328 were given empagliflozin and 341 were given a placebo [8-11].

Significant reductions in body weight, systolic blood pressure, and uric acid were seen in the empagliflozin group [8-10]. The EMMY study demonstrated that early SGLT2 inhibitor empagliflozin administration after an acute MI improves indices of cardiac function and structure, including natriuretic peptide levels. This supports the use of empagliflozin in heart failure (HF) associated with a recent MI [9]. Significant attenuations in the alterations in protein levels, soft lean mass, and skeletal muscle index were seen in the empagliflozin group [11]. Overall, empagliflozin, notably in those with baseline eGFR of 60 mL/min/1.73 m2, reduced functional deterioration in individuals with acute MI and type 2 diabetes mellitus [9].

It has been proposed that higher mortality after MI and aberrant heart rate turbulence (HRT) are related [8]. Consequently, empagliflozin enhanced the surrogate indicators for lethal arrhythmias and sudden death, heart rate variability (HRV), and HRT [8]. Patients with acute MI benefit from SGLT2 inhibitors' effects on fluid balance and improved HF [11]. Table 1 summarises our findings.

**Table 1:** Studies analysed in the review with a focus on ejection fraction, renal function, and main outcomes RCT: randomized controlled trial; T2DM: type 2 diabetes mellitus; HF: heart failure; LVEF: left ventricular ejection fraction; NT-proBNP: N-terminal pro-brain natriuretic peptide; NYHA: New York Heart Association; eGFR: estimated glomerular filtration rate; LVEDV: left ventricular end-diastolic volume; ESV: end-systolic volume.

Source	Study type and sample size	Episodes of HF and ejection fraction	Renal function	Main outcome
1. Dirk von Lewinski et al. [8]	RCT. Total = 476 predominantly with T2DM. 237 received 10 mg empagliflozin vs. 239 received a placebo	Hospitalised for HF 3/237 – empagliflozin vs. 4/239 - placebo. LVEF was 1.5% higher in the empagliflozin group than in the placebo group	No renal injury was noted between the two groups	Greater median NT-proBNP reduction in the empagliflozin group than with placebo over 26 weeks
2. Yu Hoshika et al. [9]	RCT. Total = 96. 46 received empagliflozin vs. 50 received a placebo	Similar characteristics between both groups, including LVEF and NYHA class	After 24 weeks (baseline) - (mL/min/1.73 m^2^) = empagliflozin: 64.4 ± 16.8 (64.6 ± 15.0) vs. placebo: 62.8 ± 15.4 (66.1 ± 15.7)	NT-proBNP levels were significantly decreased in both groups
3. Magnus Lundin et al. [10]	RCT. Total = 42. 20 received 25 mg empagliflozin/day vs. 22 received a placebo on top of ongoing therapy	No episodes of HF were mentioned, and LV function was not hugely affected by empagliflozin	Change in eGFR (ml/min/1.73 m^2^) = empagliflozin: 0.0 ± 5.3 vs. placebo: 1.5 ± 5.6	NT-proBNP = empagliflozin: 156 ± 295 vs. placebo: 26 ± 263. Empagliflozin did not affect LVEDV, LV systolic or mass index coronary flow reserve, ECV or aortic pulse wave velocity
4. Yu Hoshika et al. [11]	RCT. Total = 55. 25 received 10 mg empagliflozin vs. 30 received a placebo two weeks after acute myocardial infarction onset	Systolic BP = empagliflozin: 130.2 ± 1.0 mm Hg vs. placebo: 123.0 ± 13.6 mm Hg. Left ventricular ejection fraction (LVEF) = empagliflozin: 58.2 ± 11.2% vs. placebo: 47.5 ± 13.7%. The baseline LVEF of the empagliflozin group was lower than that of the placebo group	eGFR was significantly decreased only in the placebo group	-

Discussion

Experimental evidence shows empagliflozin can lead to a reduction in infarct size and therefore reduced remodelling and development of HF following MI [12]. The reason for this finding is likely multifactorial, including potential anti-inflammatory mechanisms, direct interaction with cardiomyocytes, improved myocardial energetics, activation of cardioprotective downstream mechanisms preventing remodelling processes, antifibrotic and antiapoptotic processes, and delayed progression of diabetes [12]. Thus, ischemia events ordinarily cause cell damage and MI may be resisted by the heart [12].

**Figure 2:**
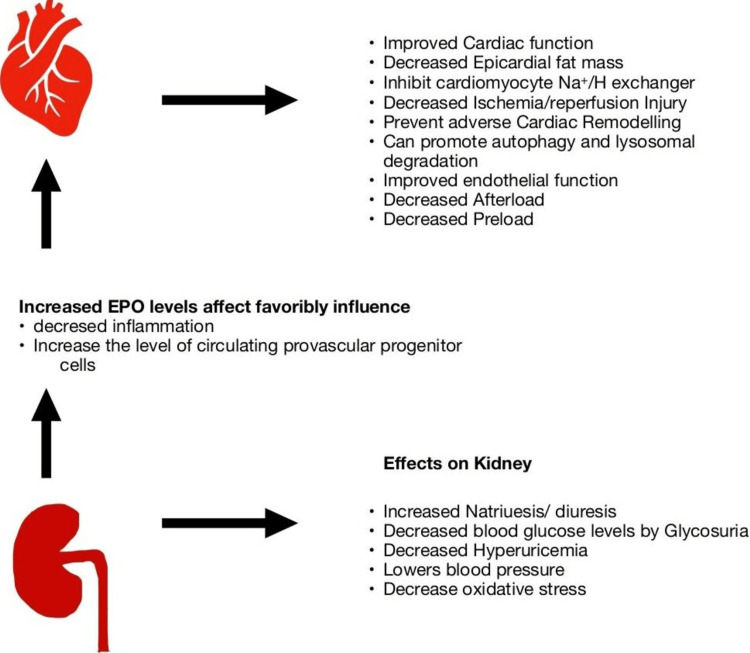
Effects of SGLT2 inhibitors on the heart and kidneys SGLT2: sodium-glucose cotransporter 2; EPO: erythropoietin. Original work and authors' own creation.

HF incidences reduce among those using empagliflozin for a short period of time who have diabetes and cardiovascular disease, yet the precise mechanism through which SGLT-2 inhibitors have a discernible beneficial effect on cardiovascular events is uncertain [4,5,13,14]. Induced osmotic diuresis, improved sodium excretion, a reduction in interstitial oedema, and improved myocardial energetics are a few explanations for the cardiovascular benefits [14]. Reduced blood pressure, weight reduction, and a retained left ventricular ejection fraction (LVEF) are further consequences that might compound over time [13,15,16]. Empagliflozin significantly reduced left ventricular (LV) end-diastolic volume and LV end-systolic volume in 84 HF patients without T2DM and with lower LVEF, according to a recent randomised study by Santos-Gallego et al. [17]. It was also linked to a decrease in LV mass [17]. Additionally, empagliflozin significantly decreases LV volume, reduces LV hypertrophy, and improves LVEF compared to placebo as well as reduced interstitial myocardial fibrosis, and reduced aortic stiffness, which enhances the quality of life of HF patients [18,19]. Some of the known effects of SGLT2 inhibitors can be shown in Figure 2 below.

The influence of SGLT2 inhibitors on NT-proBNP concentrations in HF studies is variable among different cohorts, indicating a 13% substantially decreased NT-proBNP concentration after 52 weeks compared with placebo in patients with HF. This is despite a significant improvement in LV mass [20-23]. The NT-proBNP concentrations fell in both the empagliflozin group and the control group in the EMMY/EMBODY research, which looked at how empagliflozin medication affected post-MI sympathomimetic activity [3,8]. In terms of prognostic indicators like the risk of sudden cardiac death and overall mortality, it has been shown that LVEF recovery in the weeks after a myocardial infarction is preferable to baseline LVEF alone [24,25].

From our knowledge, there are also two major clinical trials to be published soon on this topic, which should give a better idea and prove the use of empagliflozin post MI.

Limitations

This study's primary limitations include the retrospective examination of the case studies. Control case studies have to be conducted as well to support and strengthen our findings. To evaluate the long-term outcomes, more clinical trials and meta-analyses are required. Our publication highlights current research, but there are two double-blinded clinical trials upcoming soon.

## Conclusions

Studies show that in patients who recently experienced a myocardial infarction, empagliflozin was linked to a much larger NT-proBNP decrease as well as a significantly improved set of structural and functional echocardiographic measures. A substantially larger median NT-proBNP decrease was shown with empagliflozin than with placebo among patients who were hospitalised with an acute big MI when empagliflozin was started early and administered in addition to the post-MI care suggested by guidelines. Empagliflozin also reduces ejection fraction, as well as renal function. Empagliflozin significantly decreases a person's body weight, systolic blood pressure, and uric acid. Empagliflozin is effective in treating HF connected to a recent MI, according to the EMMY research. Empagliflozin shows notable attenuations in the changes in protein levels, soft lean mass, and skeletal muscle index. Overall, empagliflozin decreased functional decline in those with acute MI and type 2 diabetes mellitus, especially in those whose baseline eGFR was 60 mL/min/1.73 m2. Empagliflozin have positives on patients with post MI but upcoming trials would prove or would show the efficacy and benefits of taking empagliflozin post MI.
